# Qing Re Liang Xue Decoction Alleviates Hypercoagulability in Kawasaki Disease

**DOI:** 10.1155/2015/864597

**Published:** 2015-10-18

**Authors:** Jiao-yang Chen, Ji-ming Yin, Zhong-dong Du, Jing Hao, Hui-min Yan

**Affiliations:** ^1^Department of Pediatrics, Beijing Children's Hospital, Capital Medical University, Beijing 100045, China; ^2^Research Department Beijing Institute of Hepatology, Beijing Youan Hospital, Capital Medical University, Beijing 100045, China

## Abstract

*Objective*. Kawasaki disease (KD) is a multisystemic autoimmune vasculitis. Intravenous immunoglobulin (IVIG) is the first-line treatment for KD. It is unclear whether traditional Chinese medicine (TCM) has an effect on KD. We aimed to observe the clinical efficacy of TCM on acute KD via serum interleukin-33 (IL-33) and tumor necrosis factor alpha (TNF-*α*) measurements. *Methods*. Thirty-one KD patients were treated with Qing Re Liang Xue decoction and Western medicine (integrative medicine treatment group), while 28 KD patients were treated with Western medicine only (Western medicine treatment group). Thirty patients were included in a febrile group and 28 healthy children were included in the control group. Clinical characteristics and laboratory findings were gathered and compared. Serum IL-33 and TNF-*α* levels were measured by multiplex Luminex assay. *Results*. The platelet count in the integrative medicine treatment group was significantly lower than that in the Western medicine treatment group. The integrative medicine group had a shorter fever duration and lower IL-33 and TNF-*α* levels than those in the Western medicine group, but there were no significant differences between the two KD groups after treatment. *Conclusion*. Qing Re Liang Xue decoction improved the hypercoagulable state of KD patients. Potential myocardial protective effects require further research.

## 1. Introduction

Kawasaki disease (KD) is an acute, self-limiting multisystem inflammatory vasculitis that occurs in children. More than 85% of patients are younger than 5 years old. Although children are treated with large doses of intravenous immunoglobulin (IVIG), as many as 3–5% suffer coronary artery lesions (CAL) [1]. The etiology of KD is unknown, but the most widely proposed theories include environmental toxin exposure, autoimmune pathogenesis, and infectious diseases [2].

Cytokines like tumor necrosis factor alpha (TNF-*α*), an inflammatory mediator, are believed to be involved in the vascular lesions of KD. TNF-*α* expression is increased in the peripheral blood of KD patients during the acute phase [3] and upregulates the expression and activities of matrix metalloproteinases [4]. Interleukin-33 (IL-33), a novel member of the IL-1 family, has recently been implicated in several inflammatory and autoimmune diseases, including atherosclerosis, sepsis, asthma, allergy, Crohn's disease, ankylosing spondylitis, arthritis, and systemic lupus erythematosus [5, 6]. IL-33 is widely expressed in many tissues such as the lung, liver, central nervous system, and multiple types of cells including epithelial cells, endothelial cells, smooth muscle cells, macrophages, and fibroblasts [6–8]. IL-33 is stimulated by signals like inflammation and is secreted into the extracellular milieu [9]. IL-33 induces cytokine synthesis and mediates inflammatory responses through its receptor, ST2 [7]. KD may be induced by one or more known or unknown microbes, monocytes, macrophages, or T and B cells, which generate a systemic inflammatory response mediated by cytokines and chemical factors.

The first-line treatment of KD is IVIG and oral aspirin. IVIG reduces the prevalence of coronary artery abnormalities by reducing tissue inflammation and immune activation [10]. However, IVIG is expensive, and it can cause Qi and Yin deficiency in later stage of KD. Chinese herbs have a significant effect on some pediatric diseases. Therefore, traditional Chinese treatment might provide a new therapy for KD. We aimed to observe the clinical efficacy of Qing Re Liang Xue decoction on serum IL-33 and TNF-*α* levels in KD patients to observe whether Chinese herbs could alleviate inflammation.

## 2. Materials and Methods

### 2.1. Patients and Sample Preparations

Fifty-nine patients diagnosed with KD, 28 healthy children, and 30 febrile children (including bronchitis and pneumonia) were enrolled in the study at Beijing Children's Hospital, China. KD patients were divided into two groups with the random number table method: integrative medicine treatment group (IG) (*n* = 31) and Western medicine treatment group (WG) (*n* = 28). All KD patients met the Diagnostic Guidelines established by the Kawasaki Disease Research Committee in Japan [11]. All 59 KD patients were also diagnosed with flaring heat in qifen and yingfen, according to traditional Chinese medicine (TCM) diagnostic criteria. Exclusion criteria included incomplete KD, fever time exceeding 7 days, treatment with IVIG before hospitalization, and not having flaring heat in qifen and yingfen syndrome.

All KD patients received both IVIG (2 g/kg) and aspirin (30 mg/kg/day). The aspirin dosage was decreased to 5 mg/kg per day after normalization of C-reactive protein (CRP) values. The IG also received Qing Re Liang Xue decoction. No response to initial treatment with IVIG was defined as a fever (*T* > 38°C) lasting more than 36 hours after the end of the IVIG infusion or recurring fever after fever abatement with at least one of the clinical features of KD. Patients not responding to IVIG received additional IVIG (1 g/kg) or prednisone (1 mg/kg).

Serum was obtained from patients in the acute phase of KD before IVIG administration and in the recovery phase (7 days after fever abatement). Serum samples from healthy children and febrile patients were also collected. Patients in the febrile group had a fever before treatment. All serum samples were stored at −80°C until the assay was performed.

### 2.2. Syndrome Differentiation Treatment

The IG was treated with Qing Re Liang Xue decoction. The decoction is composed of Shengshigao (gypsum fibrosum) 15 g, Zhimu (*Rhizoma Anemarrhenae*) 9 g, Jinyinhua (*Flos Lonicerae*) 6 g, Lianqiao (*Fructus Forsythiae Suspensae*) 6 g, Huangqin (*Radix Scutellariae Baicalensis*) 9 g, Danshen (*Radix Salviae Miltiorrhizae*) 9 g, Zhuye (*Folium Phyllostachydis Henonis*) 3 g, Mudanpi (*Cortex Moutan Radicis*) 9 g, Dihuang (*Radix Rehmanniae*) 8 g, Zhizi (*Fructus Gardeniae*) 6 g, and Lingyangjiao (*Cornu Saigae Tataricae*) 0.15 g. All Chinese herbs were decocted in water and administered orally twice a day, with 7 days in a course.

### 2.3. Assay for Serum IL-33 and TNF-*α* Level

The levels of serum cytokines IL-33 and TNF-*α* were measured with a multiplex Luminex assay (EMD Millipore Co., Billerica, MA, USA), following the manufacturer's instructions. All samples were measured in duplicate as previously described.

### 2.4. Statistical Analysis

All data were analyzed using SPSS 17.0 (IBM Institute, Armonk, NY, USA) software. All data are expressed as mean ± SD. KD patients, febrile patients, and healthy children were compared using the Mann-Whitney *U* test for numerical data. Changes in serum IL-33 and TNF-*α* levels before and after IVIG treatment were compared with the paired Student's *t*-test. All *P* values were two-tailed. A *P* value less than 0.05 was considered statistically significant for all tests.

## 3. Results

### 3.1. Clinical Characteristics of KD Patients, Febrile Patients, and Healthy Children

In this study, 31 KD patients treated with integrative medicine (aged 7 months to 5 years), 28 KD patients treated with Western medicine (aged 4 months to 8 years), 30 febrile patients (aged 6 months to 7 years), and 28 healthy children (aged 5 months to 6 years) were evaluated. The clinical characteristics of all study subjects are shown in Table 1.

### 3.2. Changes in Clinical Manifestations of Different Treatment Groups

**Figure 1:**
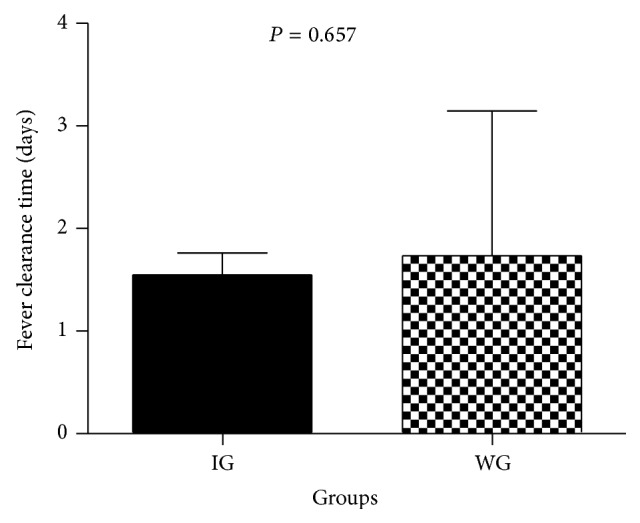
Fever clearance time in the integrative medicine group and Western medicine group. IG: integrative medicine treatment group (treated with Qing Re Liang Xue decoction and Western medicine); WG: Western medicine treatment group (treated with IVIG and/or aspirin). Data are presented as the mean ± SEM. No significant difference was observed between the two groups (*P* > 0.05).

**Figure 2:**
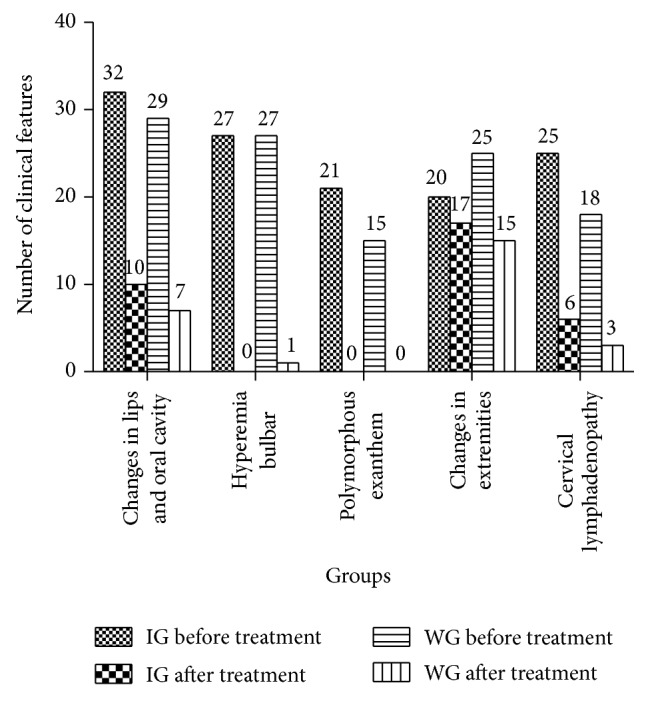
Comparison of numbers of clinical symptoms between the integrative medicine group and Western medicine group before and after therapy. IG: integrative medicine group (treated with Qing Re Liang Xue decoction and Western medicine); WG: Western medicine treatment group (treated with IVIG and/or aspirin).

Temperature recovery time in the IG was 1.55 ± 1.16 days, which was shorter than that of the WG (1.79 ± 1.44 days). However, there was no significant difference between the two groups (*P* = 0.657) (Figure 1). Changes in lips and oral cavity appearance, hyperemia bulbar, polymorphous exanthema, changes in extremities, and cervical lymphadenopathy are the main symptoms of KD. The occurrence of these symptoms was obviously decreased after treatment in both IG and WG, but there was no significant difference between the two groups (Figure 2).

### 3.3. Laboratory Examination of IG and WG before and after Therapy

White blood cells (WBC), platelets (PLT), C-reactive protein (CRP), and erythrocyte sedimentation rate (ESR) were evaluated in all patients. WBC, ESR, and CRP were lower after treatment, and PLT was higher in both the IG and WG. There were significant differences in WBC, PLT, and CRP between before and after treatment in each group. After treatment, PLT in the IG was significantly lower than that in the WG (*P* = 0.048) (Table 2).

### 3.4. Comparison of Serum IL-33 and TNF-*α* Levels among Groups

**Figure 3:**
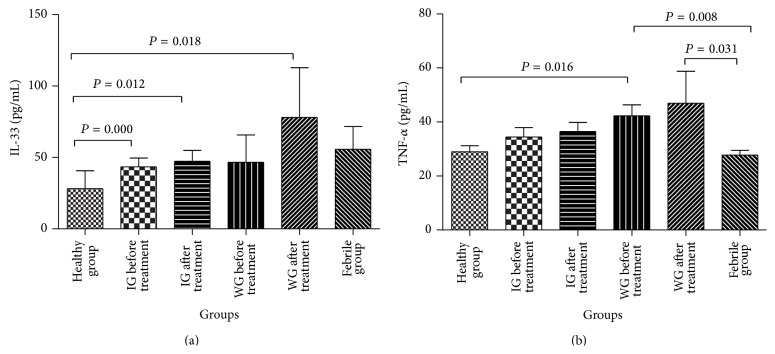
Serum IL-33 and TNF-*α* levels among groups. IL: interleukin; TNF: tumor necrosis factor; IG: integrative medicine treatment group (treated with Qing Re Liang Xue decoction and Western medicine); WG: Western medicine treatment group (treated with IVIG and/or aspirin). Data are presented as the mean ± SEM. Significant differences comparing IL-33 level in the healthy group with the IG before and after treatment and the WG after treatment are marked as *P* < 0.05. Significant differences comparing TNF-*α* levels in the WG before treatment with the healthy and febrile groups and the WG after treatment with the febrile group are marked as *P* < 0.05.

Levels of IL-33 and TNF-*α* in serum were detected in healthy children, febrile children without KD, and KD patients before and after treatment.  Figure 3 shows that the serum levels of IL-33 in healthy controls (28.1 ± 56.0 pg/mL) were significantly lower than those in IG before therapy (43.4 ± 29.0 pg/mL) and after therapy (47.3 ± 36.6 pg/mL) and WG after IVIG (78.0 ± 143.7 pg/mL) (*P* < 0.05) (Figure 3(a)). There were no significant differences in IL-33 levels between the KD groups and the febrile group.

TNF-*α* levels in the WG before therapy (42.3 ± 21.1 pg/mL) were significantly higher than those of healthy controls (29.0 ± 11.3) and febrile children (27.8 ± 9.3 pg/mL). After therapy, TNF-*α* level increased in the WG (47.0 ± 57.8 pg/mL). TNF-*α* levels were significantly higher in the WG than those in the febrile group (27.8 ± 9.3 pg/mL) (Figure 3(b)). No significant differences were observed in TNF-*α* level between the IG before and after treatment and healthy group or febrile patients.

IL-33 and TNF-*α* in the IG were lower than those in the WG (47.3 ± 36.5 pg/mL versus 78.0 ± 143.7 pg/mL and 36.5 ± 18.4 pg/mL versus 47.0 ± 57.8 pg/mL, resp.), but the differences were not significant (Figures 3(a) and 3(b)).

## 4. Discussion

In this study, we found that treatment with Qing Re Liang Xue decoction plus Western medicine resulted in a significantly lower increase in PLT levels than in the Western medicine group. Moreover, inflammatory cytokines were lower in KD patients treated with integrative medicine compared with Western medicine, although the differences were not significant. We used multiplex Luminex assay because it offers a highly sensitive and accurate method of determining serum levels in samples and uses small quantities.

KD patients treated with integrative medicine had a normal temperature more quickly than those in the Western medicine group, although the difference was not significant. Therefore, Chinese medicine could possibly abate the fever in KD patients. In Qing Re Liang Xue decoction,* Cornu Saigae Tataricae*, gypsum fibrosum,* Radix Rehmanniae*, and* Cortex Moutan Radicis* have cold properties according to TCM theory, which might have a role in removing heat to cool the blood and drop temperature.

In the laboratory tests after treatment, only the PLT level was significantly different between the two KD groups. Platelet counts are usually evaluated after day 7 of an illness [12], which makes the patients hypercoagulable. Coronary artery lesions (CAL) are the most common and worst complication of KD and are related to cardiac disease progression in adults who have a KD history. One risk factor for predicting aneurysms is platelet count. The smaller increase in PLT levels in the IG compared with the WG might be caused by the* Radix Salviae Miltiorrhizae* and* Cortex Moutan Radicis*. These two herbs can activate circulation to remove blood stasis, according to TCM.* Radix Salviae Miltiorrhizae* can act as an antiarrhythmic, antiplatelet aggregate and has myocardial protective effects [13]. Qingying decoction and Qingwenbaidu decoction are classical Chinese medicine formulae to clear heat. Qingying decoction is supposed to clear heat and cool blood and can act as an antipyretic, sedative, anti-inflammatory, and antibiotic and can repair myocardial cells [14]. Modern pharmacological study indicates that heat-clearing and blood-activating Chinese medicines could improve microcirculation, increase hemodynamics, reduce platelet aggregation, and prevent thrombus formation. Qing Re Liang Xue decoction gathered the advantages of many prescriptions; therefore, we hypothesize that Qing Re Liang Xue decoction could relieve hypercoagulability and decrease CAL incidence, but more research is required.

IL-33 expression was significantly higher in KD patients compared with healthy control patients, but there was no significant difference between the two KD groups and febrile group. TNF-*α* in the WG before treatment was significantly higher than that in the healthy and febrile groups. TNF-*α*, either alone or in cooperation with other cytokines, regulates the production of chemokines on endothelial cells [3]. IL-33 is a newly reported cytokine in the IL-1 family and can be produced by various types of tissues and cells. It has been reported to participate in the development and progression of many diseases like rheumatoid arthritis, systemic lupus erythematosus, ankylosing spondylitis, Behçet's disease, systemic sclerosis, and coronary artery disease [6, 15]. IL-33 functions by binding to its receptor ST2 as a proinflammatory cytokine [16] and shares the IL-1R accessory protein (IL-1RAcP) and subsequent IL-1RAcP-dependent signaling pathway activation with other members of the IL-1 family [17]. In our study, serum IL-33 levels were higher in the IG and WG after treatment, although the differences were not significant. Nevertheless, this slight effect might suggest that an inflammatory response was present. Immune disorder and inflammatory cytokine activity increase in the middle stages of KD, from the 3rd to 5th weeks. During this period, patients are at high risk of CAL development. Therefore, inhibiting this acute inflammation is important for reducing the CAL risk. Serum IL-33 levels in the IG were lower than the WG after treatment, and fever duration in the IG was shorter than that in the WG, although these differences were not significant. These results indicate that Chinese herbs might help prevent the progression of KD and possibly protect the myocardium.

Many studies have focused on the relationship between IL-33 and TNF-*α*. Previous studies reported that TNF-*α* could stimulate the production of IL-33 [18]. In the synovial fibroblasts of patients with rheumatoid arthritis, IL-33 was detected and was markedly upregulated by the addition of TNF-*α* and IL-1*β* [19]. Matsuyama et al. also found that IL-33 expression was induced by IL-1*β*, and IL-33 expression was enhanced by costimulation with IL-1*β* and TNF-*α* [20].

Recent studies have shown that the inflammatory factors IL-6, TNF-*α*, C-reactive protein, adhesion molecules, and Endothelin-1 participate in the formation of blood stasis directly or indirectly [21]. Some studies have previously reported that TNF-*α* is closely linked to inflammatory responses and the development and maintenance of arterial remodeling through the release of cytokines [22]. TNF-*α* appears to be critical during the evolution of coronary artery damage in a murine model of KD [3], and chronic overexpression of TNF-*α* in the heart resulted in dilated cardiomyopathy and increased mortality [23]. Xie et al. found that the expressions of apolipoprotein A-I and *α*_1_-antitrypsin were different in patients with blood stasis syndrome and coronary heart disease (CHD) with unstable angina pectoris. This result indicated that inflammation may be one of the mechanisms of blood stasis syndrome in CHD [24].* Radix Salviae Miltiorrhizae* can improve blood stasis and reduce IL-6 levels [25]. KD patients have severe inflammation, which can cause myocardial damage. However, some Chinese herbal medicines can activate blood and remove stasis to treat inflammation and myocardial injury. This mechanism could explain why IL-33 and TNF-*α* did not decrease after treatment in the two KD groups.

Our study has some limitations. The small sample size was not adequate for reliable assessment. The prevalence of KD has regional and ethnic differences, so a larger sample size from different populations is required to replicate the results. Furthermore, we did not detect cellular IL-33 and TNF-*α* levels. Serum IL-33 and TNF-*α* levels are relatively low and were occasionally out of range for measurement. Measuring cellular cytokine expression could contribute more to the role and origin of IL-33 and TNF-*α* during KD progression. In this study, we collected serum from KD patients on the 1st and 7th days to observe changes in serology. There was no significant difference from before to after treatment in serum IL-33 or TNF-*α* levels in either treatment group. This could be because of the short surveillance time. KD requires a long rehabilitation and the cytokine levels could change over time. A follow-up evaluation might be important in patients with KD. Finally, the decoction has an unfavorable taste for younger children, so compliance in the study was poor. Innovation in Chinese traditional drug agents could help improve this issue, but further study is required.

## 5. Conclusion

Qing Re Liang Xue decoction reduced platelet count, which indicates that the decoction could alleviate hypercoagulability in KD patients. Serum IL-33 and TNF-*α* levels were remarkably elevated in KD patients, which indicates that KD development involves inflammation. Qing Re Liang Xue decoction could decrease serum IL-33 and TNF-*α* levels. Therefore, the traditional Chinese medicine might have an effect on alleviating inflammation and protecting the myocardium in KD patients.

## Figures and Tables

**Table 1:** Clinical characteristics of KD patients, febrile patients, and healthy children.

	IG (*n* = 31)	WG (*n* = 28)	Febrile group (*n* = 30)	Healthy group (*n* = 28)
Male/female	22/9	25/3	24/6	22/6
Age onset (years)	2.1 ± 1.3	2.1 ± 1.8	3.4 ± 1.7	3.6 ± 1.1
<1 year	8	8	2	3
1–5 years	23	19	24	21
>5 years	0	1	4	4

Note: IG: integrative medicine treatment group (treated with Qing Re Liang Xue decoction and Western medicine); WG: Western medicine treatment group (treated with IVIG and/or aspirin); febrile group: children who had a fever with pneumonia or bronchitis; healthy group: healthy children without any diseases; KD: Kawasaki disease.

**Table 2:** Comparison of laboratory tests in different treatment groups before and after treatment.

	Integrative medicine treatment group		Western medicine treatment group	
	Before treatment	After treatment	Before treatment	After treatment
WBC (×10^9^/L)	13.5 ± 6.3	8.7 ± 2.7^a^	14.1 ± 6.7	8.5 ± 3.1^b^
PLT (×10^9^/L)	374.9 ± 110.9	478.9 ± 138.3^a^	371.3 ± 130.2	541.5 ± 138.4^bc^
CRP (mg/L)	71.7 ± 48.2	9.0 ± 2.4^a^	67.4 ± 45.7	8.3 ± 1.2^b^
ESR (mm/h)	42.4 ± 25.6	33.7 ± 18.4	51.0 ± 30.0	35.9 ± 20.2

Note: WBC: white blood cell; PLT: platelets; CRP: C-reactive protein; ESR: erythrocyte sedimentation rate. Compared with the integrative medicine group before treatment, ^a^ *P* < 0.05; compared with Western medicine treatment group before treatment, ^b^ *P* < 0.05; compared with the integrative medicine treatment group after treatment, ^c^ *P* < 0.05.

## References

[1] Burns J. C., Glodé M. P. (2004). Kawasaki syndrome. *The Lancet*.

[2] Rowley A. H. (2011). Kawasaki disease: novel insights into etiology and genetic susceptibility. *Annual Review of Medicine*.

[3] Hui-Yuen J. S., Duong T. T., Yeung R. S. M. (2006). TNF-*α* is necessary for induction of coronary artery inflammation and aneurysm formation in an animal model of Kawasaki disease. *The Journal of Immunology*.

[4] Xue H., Sun K., Xie W.-P. (2012). Etanercept attenuates short-term cigarette-smoke-exposure-induced pulmonary arterial remodelling in rats by suppressing the activation of TNF-*α*/NF-*κ*B signal and the activities of MMP-2 and MMP-9. *Pulmonary Pharmacology &Therapeutics*.

[5] Beltrán C. J., Núñez L. E., Díaz-Jiménez D. (2010). Characterization of the novel ST2/IL-33 system in patients with inflammatory bowel disease. *Inflammatory Bowel Diseases*.

[6] Duan L., Chen J., Gong F., Shi G. (2013). The role of IL-33 in rheumatic diseases. *Clinical and Developmental Immunology*.

[7] Schmitz J., Owyang A., Oldham E. (2005). IL-33, an interleukin-1-like cytokine that signals via the IL-1 receptor-related protein ST2 and induces T helper type 2-associated cytokines. *Immunity*.

[8] Préfontaine D., Lajoie-Kadoch S., Foley S. (2009). Increased expression of IL-33 in severe asthma: evidence of expression by airway smooth muscle cells. *The Journal of Immunology*.

[9] Haraldsen G., Balogh J., Pollheimer J., Sponheim J., Küchler A. M. (2009). Interleukin-33—cytokine of dual function or novel alarmin?. *Trends in Immunology*.

[10] Galeotti C., Bayry J., Kone-Paut I., Kaveri S. V. (2010). Kawasaki disease: aetiopathogenesis and therapeutic utility of intravenous immunoglobulin. *Autoimmunity Reviews*.

[11] Ayusawa M., Sonobe T., Uemura S. (2005). Revision of diagnostic guidelines for Kawasaki disease (the 5th revised edition). *Pediatrics International*.

[12] Newburger J. W., Takahashi M., Gerber M. A. (2004). Diagnosis, treatment, and long-term management of Kawasaki disease: a statement for health professionals from the Committee on Rheumatic Fever, Endocarditis and Kawasaki Disease, Council on Cardiovascular Disease in the Young, American Heart Association. *Circulation*.

[13] Zhang M., Zhang H., Xu P., Su R.-Y. (2008). Research progress of Tanshinone II A pharmacological activity. *Yi Yao Dao Bao*.

[14] Zhang B.-G., Cheng T.-F., Liu Q.-F. (2009). Efficacy and modern clinical application of Qingying decoction. *Chinese Traditional Patent Medicine*.

[15] Tu X., Nie S., Liao Y. (2013). The IL-33-ST2L pathway is associated with coronary artery disease in a Chinese Han population. *The American Journal of Human Genetics*.

[16] Xu D., Jiang H.-R., Kewin P. (2008). IL-33 exacerbates antigen-induced arthritis by activating mast cells. *Proceedings of the National Academy of Sciences of the United States of America*.

[17] Seltmann J., Werfel T., Wittmann M. (2013). Evidence for a regulatory loop between IFN-*γ* and IL-33 in skin inflammation. *Experimental Dermatology*.

[18] Wood I. S., Wang B., Trayhurn P. (2009). IL-33, a recently identified interleukin-1 gene family member, is expressed in human adipocytes. *Biochemical and Biophysical Research Communications*.

[19] Palmer G., Talabot-Ayer D., Lamacchia C. (2009). Inhibition of interleukin-33 signaling attenuates the severity of experimental arthritis. *Arthritis and Rheumatism*.

[20] Matsuyama Y., Okazaki H., Hoshino M. (2012). Sustained elevation of interleukin-33 in sera and synovial fluids from patients with rheumatoid arthritis non-responsive to anti-tumor necrosis factor: possible association with persistent IL-1*β* signaling and a poor clinical response. *Rheumatology International*.

[21] Ma X.-J., Yin H.-J., Chen K.-J. (2007). Research progress of correlation between blood-stasis syndrome and inflammation. *Chinese Journal of Integrative Medicine*.

[22] Wright J. L., Tai H., Wang R., Wang X., Churg A. (2007). Cigarette smoke upregulates pulmonary vascular matrix metalloproteinases via TNF-*α* signaling. *American Journal of Physiology—Lung Cellular and Molecular Physiology*.

[23] Kuhota T., McTiernan C. F., Frye C. S. (1997). Dilated cardiomyopathy in transgenic mice with cardiac-specific overexpression of tumor necrosis factor-alpha. *Circulation Research*.

[24] Xie H., Wang W., Zhao H.-H., Guo S.-Z. (2010). Relationgship between plasma ApoA-I, *α*1-AT and inflammation in blood stasis syndrome of coronary heart disease. *Journal of Beijing University of Traditional Chinese Medicine*.

[25] Lv Y., Wang Y.-P., Li W.-J., Zhang L. (2006). Clinical researches that the serum NO ET IL-6 varieties of chronic renal failure patients with blood stasis symptom and effect of Lei's Danshen tablets. *Chinese Traditional Patent Medicine*.

